# Effect of predators on *Anopheles arabiensis* and *Anopheles funestus* larval survivorship in Homa Bay County Western Kenya

**DOI:** 10.1186/s12936-023-04741-w

**Published:** 2023-10-05

**Authors:** Pauline Winnie Orondo, Guofa Zhou, Kevin O. Ochwedo, Xiaoming Wang, Benyl M. Ondeto, Ming-Chieh Lee, Steven G. Nyanjom, Harrysone Atieli, Andrew K. Githeko, James W. Kazura, Guiyun Yan

**Affiliations:** 1 Department of Biochemistry, Jomo Kenyatta University of Agriculture and Technology, Nairobi, Kenya; 2 International Center of Excellence for Malaria Research, Tom Mboya University, College of Maseno University, Homa Bay, Kenya; 3 Program in Public Health, College of Health Sciences, University of California at Irvine, Irvine, CA USA; 4 Centre for Global Health Research, Kenya Medical Research Institute, Kisumu, Kenya; 5 Center for Global Health & Diseases, School of Medicine, Case Western Reserve University, Cleveland, OH USA

**Keywords:** Larval control, Predation habitat, *Anopheles*

## Abstract

**Background:**

The rise of insecticide resistance against malaria vectors in sub-Saharan Africa has resulted in the need to consider other methods of vector control. The potential use of biological methods, including larvivorous fish, *Bacillus thuringiensis israelensis* (Bti) and plant shading, is sustainable and environmentally friendly options. This study examined the survivorship of *Anopheles arabiensis* and *Anopheles funestus* larvae and habitat productivity in four permanent habitat types in Homa Bay county, western Kenya.

**Methods:**

Predator densities were studied in a laboratory setup while habitat productivity and larval survivorship was studied in field setup.

**Results:**

Fish were observed as the most efficient predator (75.8% larval reduction rate) followed by water boatman (69%), and dragonfly nymph (69.5%) in predation rates. Lower predation rates were observed in backswimmers (31%), water beetles (14.9%), water spiders (12.2%), mayflies (7.3%), and tadpoles (6.9%). Increase in predator density in the field setup resulted in decreased *Culex* larval density. Larval and pupa age–specific distribution was determined and their survivorship curves constructed. Combined larvae (Stage I–IV) to pupa mortality was over 97% for *An. arabiensis* and 100% for *An. funestus*. The highest larval stage survival rate was from larval stages I to II and the lowest from larval stage IV to pupa. Stage-specific life tables indicated high mortality rates at every developmental stage, especially at the larval stage II and III.

**Conclusion:**

Determination of the efficiency of various larval predators and habitat productivity will help with the correct identification of productive habitats and selection of complementary vector control methods through environmental management and/or predator introduction (for instance fish) in the habitats.

**Supplementary Information:**

The online version contains supplementary material available at 10.1186/s12936-023-04741-w.

## Background

Malaria remains a major public health concern in sub-Saharan Africa despite major control advances in many countries. Africa still suffers from the highest morbidity and mortality rates with over 90% of all malaria cases globally being reported from the continent [1]. The burden of this disease has persisted due to challenges including parasite drug resistance [2, 3], residual transmission [4], and mosquito insecticide resistance [5, 6] thus hampering malaria eradication in Africa. With the recent introduction of the malaria vaccine in children under the age of 5 years [7]; which is one of the most vulnerable groups affected by malaria, a further decrease in malaria deaths is anticipated [8]. This coupled with timely effective malaria treatment and vector control measures should reduce reported malaria cases.

Recent interest in the control of immature malaria vectors has been hailed as a potential for vector control in certain vector habitats like abandoned goldmines and rice paddies [9–11]. This has been proposed to be potentially effective as it targets mosquitoes confined in their aquatic habitats before dispersal and feeding on humans and other hosts. Understanding survivorship and mortality of immature *Anopheles* mosquitoes and their relationship with other macroinvertebrates in the aquatic habitats can contribute towards larval control strategies. *Anopheles arabiensis*, a major malaria vector in Africa, is known to inhabit specific habitat types which include temporary small sunlit pools [12]. Recent studies have shown that habitat stability has a direct negative impact on malaria vector larval densities [12]. Temporary pools are observed to harbour more immature *An. arabiensis* [12] than the permanent and semi-permanent habitats, which are often inhabited with more predators [13], thus ovipositing females prefer temporary pools to more stable ones for the sake of the survival of the immature into adults [14]. This is in addition to other oviposition selection site cues preferred by the gravid mosquito for larval survival and maturation.

Larval survivorship and maturation in the aquatic habitats are highly dependent on the stability of the aquatic habitats. These aquatic habitats are a host to a variety of predatory and symbiotic insects. These predators play a major role in the regulation of vector populations before they emerge as adults. Studies have shown that several aquatic predator species prey on mosquito immature stages including Coleopterans, Amphibians, Hemipterans, Odonates, fishes, Arachnids, and Ephemeropterans [15, 16]. These species affect larval densities, survival and maturation thus affecting adult mosquito populations and competency. In addition to predator populations, studies have shown that the densities of the vectors within a habitat affect the final habitat productivity of adult vectors [17]. It has been observed that the higher the density of larvae in a habitat the less productive the habitat is as compared to one that is not densely populated.

This study examined age-specific survivorship of *An. arabiensis* and *Anopheles funestus* in permanent habitats in Homa Bay County, western Kenya. The abundance and contribution of potential larval predators was also tested in the laboratory and in their natural environment.

## Methods

### Study site

This study was conducted in Homa Bay County, Western Kenya, along the shores of Lake Victoria (34.6°E and 0.5°S; 1330 m above sea level). The mean annual maximum temperature of the area is approximately 30 ℃ and the mean annual rainfall is around 1100 mm, with two rainy seasons. This area has been previously described in details by previous studies [12, 18–20]. Briefly, the area has been modified by the construction of concrete-based irrigation canals to increase household food production. The locals engage in animal husbandry, crop and fish farming. This area is known to be a malaria endemic area with malaria transmission occurring throughout the year. The main malaria vectors are *An. arabiensis* and to a lesser extent, *An. funestus* [19].

### Laboratory predator experiment

This experiment was conducted following Allo and Mekhlif [21] methods, with slight modifications. The following predators were used in the study: Coleoptera (beetles), Amphibians (tadpoles), Hemiptera (boatmen, backswimmers, and water scorpions), Odonata (damselfly nymphs and dragonfly nymphs), fish (*Gambusia affinis*), Arachnida (spiders), and Ephemeroptera (Mayflies). These predators were not identified to species level except for the fish. A basin with no predator was also included as a control. The experiments were carried out on a daily basis, with the predators and mosquito larvae being replaced-every-24 h for fit and uncompromised samples. Larval stages II and III were introduced in predator- inhabited larval basins at 1:20 ratio, with a max of 5 predators and 100 larvae. The tops of the basins were covered with a netting material which was secured with rubber bands to prevent the predators from escaping, oviposition from other wild mosquitoes, and larval predation by other predators. The predators were not fed prior to the commencement of each experiment as they were collected daily from their natural habitats and introduced in the experimental basins. The experimental basins were placed on the bench in the insectary work space with free air flow. After 24 h, larval counts were performed. A minimum of 3 replicates were performed for each predator.

### Vertical life tables: field experiment

The survivorship and mortality of *An. arabiensis* and *An. funestus* were determined using vertical life tables in the natural habitats. A vertical life table was created using natural habitats that allow for continuous oviposition resulting in overlapping generations, as opposed to a horizontal life table which is used for a cohort of habitats that are followed over time until a single generation is exhausted [14, 22, 23]. Four permanent habitat types were chosen for follow-up, which included six replicates of rice paddies, drainage ditches, fish ponds, and five replicates of man-made ponds. Larval sampling was conducted daily for 29 days in September and October of 2019. Larval species and densities of various larval stages, as well as predator types and densities, were recorded after sampling using standard dipping method.

### Data analysis

Raw field data was entered into data forms and later entered into MS Excel. JMP Pro Ver. 16 software was used to analyse the data. Larval reduction rates in predator laboratory experiments and the predator—larvae relationship in the field experiment were calculated using generalized regression with Poisson distribution. To determine the larval survival rates in the life table analysis, the overall average daily survival rates from one larval stage to the next was determined for both *An. arabiensis* and *An. funestus* in the different habitats. Five-day smoothed dynamics of different stages of mosquitoes by species and habitat types was also determined.

## Results

### Predator experiment

**Fig. 1:**
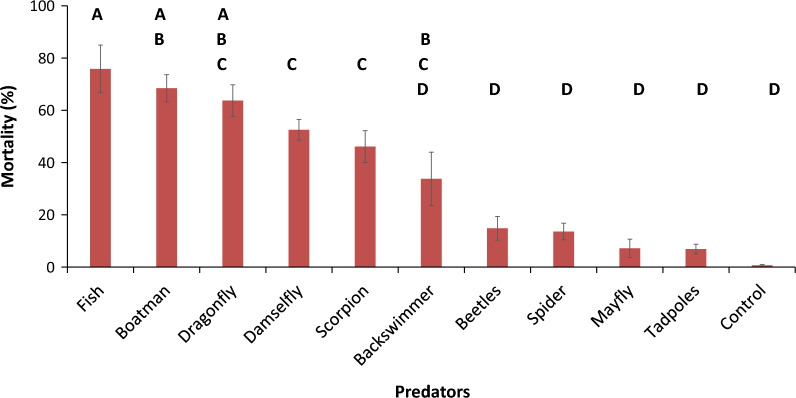
Predation rates *An. arabiensis* larvae in experimental basins in a laboratory set-up

The experiment used a predator-to-larvae ratio of 1:20 with each experimental basin containing 3 to 5 predators. A significant predation effect (F = 35.5, P < 0.0001) on larval numbers (Table 1) was observed within 24 h. Among the predators studied, predation by fish resulted in the greatest larval reduction (75.8%; F = 40.98, P < 0.0001) in the experimental basins. However, when fish (75.8%; F = 40.98, P < 0.0001), boatman (69%; F = 33.59, P < 0.0001), and dragonfly nymph (69.5%; F = 28.83, P < 0.0001) reduction rates were compared, there was no significant difference in their predation rates. Dragonfly nymph (69.5%; F = 28.83, P < 0.0001), damselfly nymph (52.4%; F = 17.68, P < 0.0001), and water scorpion (49.8%; F = 11.25, P = 0.0145) were also similar in predation rates. Water beetles (14.9%; F = − 20.03, P = 0.0001), water spiders (12.2%; F = − 21.27, P < 0.0001), mayflies (7.3%; F = − 27.72, P < 0.0001), and tadpoles (6.9%; F = − 27.99, P < 0.0001) were found to be poor predators, with larval reduction rates that were not significantly different from the control basins. Backswimmers (31%; F = − 1.11, P = 0.92) were deemed average larval consumers because their consumption was within the median range (Fig. 1).

**Table 1:** Summary results of the model fit of the predator laboratory experiment

Predator	Number of predators used	Estimate	Std Error	t Ratio	P-value	95% Confidence interval
*Gambusia affinis* fish (Family Poeciliidae, order Cyprinodontiformes)	65	40.98	5.38	7.61	< 0.0001	51.6—> 30.4
Water boatman (Family Corixidae, Order Hemiptera)	111	33.59	3.88	8.65	< 0.0001	41.2—> 25.9
Dragonfly nymph (Family Corduliidae, Order Odonata)	22	28.83	4.91	5.87	< 0.0001	38.5—> 19.1
Damselfly nymph (Family Lestidae, Order Odonata)	99	17.68	4.06	4.35	< 0.0001	25.7—> 9.7
Water scorpion (Family Nepidae, order Hemiptera)	68	11.25	4.56	2.47	0.0145	20.2—> 2.3
Backswimmer (Family Notonectidae, Order Hemiptera)	10	− 1.11	10.75	− 0.1	0.92	20.1- > − 22.3
Water beetles (Family Hydrophilidae and Dytiscidae, Order Coleoptera)	71	− 20.03	5.05	− 3.97	0.0001	− 10.1—> − 30.0
Water spiders (Family Dictynidae, Order Araneae)Spider	30	− 21.27	4.37	− 4.87	< 0.0001	− 12.7—> − 29.9
Mayfly (Family: Baetidae Order: Ephemeroptera)	26	− 27.72	5.38	− 5.15	< 0.0001	− 17.1—> − 38.3
Tadpoles (Class Amphibians)	108	− 27.99	4.28	− 6.53	< 0.0001	− 19.5—> − 36.4

### Habitat types

**Fig. 2:**
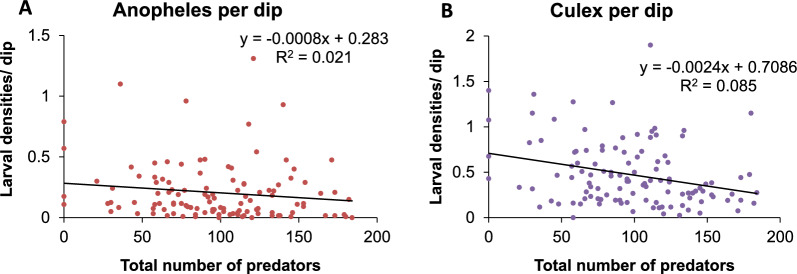
Correlation between larval densities and total number of predators for (**A**) *Anopheles* larvae and (**B**) *Culex* larvae per dip

Larval samples were collected from 23 different permanent habitats over the course of the study. These habitats were classified into four habitat types. These were 6 replicates of rice paddies, drainage ditches, fish ponds each and five replicates of man-made ponds. The rice paddies contained recently transplanted rice thus were highly flooded. The drainage ditches were used to drain excess water from the farms while the fishponds were abandoned tilapia fishponds. The man-made ponds were formed as a result of human activities such as sand and ballast harvesting. There was no association between predator and *An. arabiensis* densities when all habitats were combined (Fig. 2a) (F = 2.37, df = 1114, P = 0.127). However, there was a strong negative association between predator densities and *Culex* larvae densities, which was statistically significant (Fig. 2b) (F = 10.44, df = 1114, P = 0.0016).

### Vertical life tables

**Fig. 3:**
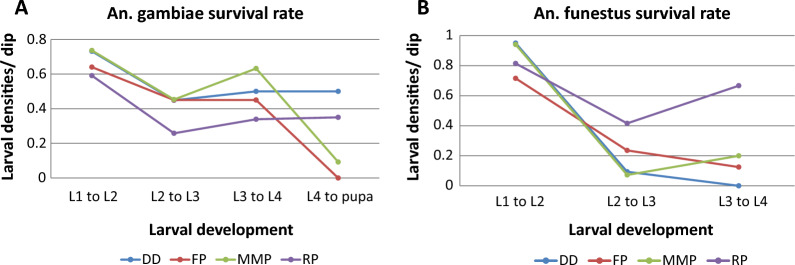
Mosquito age specific survival rate for (**A**) *An. arabiensis* and (**B**) *An. funestus* in drainage ditches (DD), fishponds (FP), man-made ponds (MMP), and rice paddies (RP) habitat types

**Fig. 4:**
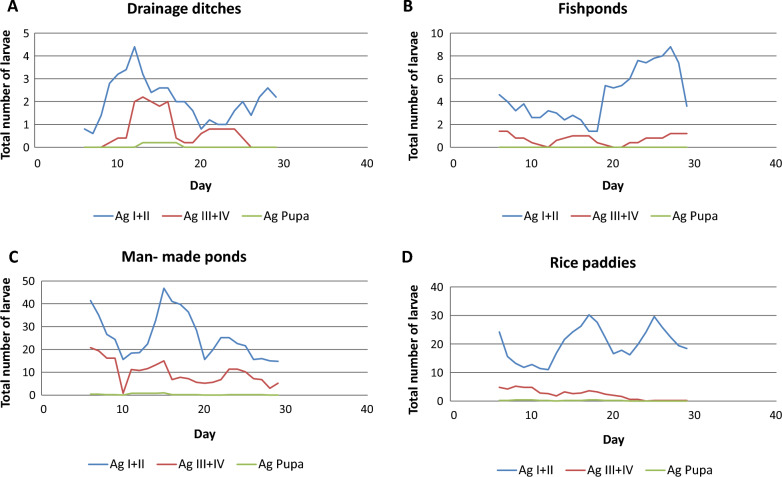
Total number of *An. arabiensis* in (**A**) drainage ditches, **B** fishponds, **C** man-made ponds, and **D** rice paddies using five- day smoothed dynamics

**Fig. 5:**
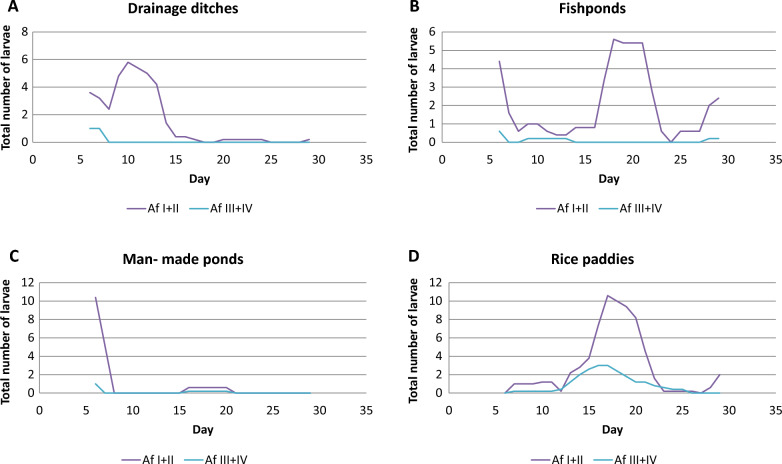
Total number of *An. funestus* in (**A**) drainage ditches, **B** fishponds, **C** man-made ponds, and **D** rice paddies using five- day smoothed dynamics

*Anopheles arabiensis* survived best in drainage ditches, while *An. funestus* thrived best in rice paddies (Fig. 3). In all habitat types, the highest larval stage survival rate was observed in both *An. arabiensis* (Fig. 3a) and *An. funestus* (Fig. 3b) species from larval stage I to II, with the lowest being larval stage IV to pupae for *An. funestus*. During the study period, no *An. funestus* pupae were collected. The mortality rates of *An. arabiensis* immatures (larval stages I–IV) were 97.9%, 100%, 98.3%, and 99.2% in drainage ditches, fishponds, man-made ponds, and rice paddies respectively while the mortality of *An. funestus* was 100% in all habitat types. Furthermore, an increase in younger larval stages resulted in an increase of older larval stages a few days and vice versa (Fig. 4). *Anopheles arabiensis* immature larval stages peaked between the 10th and 20th day in drainage ditches, man- made ponds, and rice paddies habitat types as opposed to fish ponds (Fig. 4). A 5-day smooth dynamics average of the larvae and pupae in the four habitat types showed that the age distribution of *An. arabiensis* and *An. funestus* larvae fluctuated over the study period. In the case of immature larval stages of *An. funestus*, only fish ponds and rice paddies had similar peak patterns between days 15 and 25, whereas drainage ditches peaked before day 15 (Fig. 5).

## Discussion

This study was conducted to understand age-specific survivorship of immatures of malaria vectors in permanent habitats and how predators and habitat types affect larval to pupal survivorship. Fish was observed to be the most efficient predator of all tested. A strong negative correlation was observed between predator numbers and *Culex* larvae reduction. This study observed the highest stage survival rate from larval stages I to II and the lowest from larval stage IV to pupae. An increase in younger larval stage resulted in a corresponding increase of older stages. Stage-specific life tables indicated high mortality rates at every developmental stage, especially at the larval stages II and III. Mortality was over 97% for *An. arabiensis* and 100% for *An. funestus*. Previous studies have demonstrated that mortality at various aquatic stages result from several factors including predation, cannibalism and environmental factors [24–26].

Various macroinvertebrates have been known to effectively predate on mosquitoes including Hemiptera, Coleoptera, fishes, amphibians, Odonata, and other Diptera [27, 28]. Chemical use on larval habitats for mosquito control might be effective in small habitats due to the ease of administration of the chemical and the accessibility of the aquatic habitat while use of natural predators might be effective in larger habitats [29], which are mostly not fully accessible during vector control using insecticide. Mosquito predators are more abundant in larger, older and more stable habitats due to more prey and lesser chances of the habitat drying up [29] to enable the completion of their aquatic stages. Habitat preference for oviposition by gravid mosquito species is dependent on several factors (usually visually or with the use of chemoreceptors e.g. tactile or olfactory on its legs or antennae) to determine the suitability of the habitat to sustain the larvae to maturation. Amongst these factors, a gravid mosquito usually compromises between competition from other larval species as observed in small habitats and predation which is common in large habitats [29]. Studies have demonstrated that *An. arabiensis* seeks for favourable habitats to improve their chances of survival [30], while other mosquito species avoid habitats with predators and competitors [31, 32]. However, some mosquitoes prefer habitats with conspecific larvae, as this may be indication of the suitability of the habitats [33].

Fish belonging to the *Gambusia* species performed comparably with dragonflies and water boatman. This is similar to results from previous studies which showed that fish and dragon flies resulted in significant reduction of larvae [28, 34]. Dragonflies, are known to be effective predators in semi-field conditions but are not selective feeders in natural conditions [28]. Various bugs in the order Hemiptera have also been observed to possess predatory habits stirring interest in their use as biological control agents against mosquito larvae including Notonectids, commonly known as backswimmers. These have been considered the most promising [35–37]. More recent studies have however shown that these predators are more efficient with increase prey density [21] and are more effective against late stages of larvae [38]. Therefore, low prey densities could explain the low predation rates observed in this study. Some studies have however shown that some predators (dragonfly and mayfly) are more effective against early instar larvae and predation decreases with increased larval instar stage [39].

Colonization of habitats by predators has been observed to be partly due to the presence of mosquito larvae and other inherent water physical and chemical parameters. Understanding these abiotic and biotic factors which allow for the co-existence of immature mosquitoes and predator may contribute to the control of malaria [40]. It has been demonstrated that some mosquito species avoid habitats with supposed competitors and predators [41, 42]. These results show that, in the natural set-up, increase in predator densities resulted in decline in *Culex* density. This is concurrent with other studies [43] which observed that an increase in predator abundance increased the chances for mosquito larvae being present. In contrast, there was no effect of predator densities on *Anopheles* species. This could be due to the types of habitats selected as these were permanent habitats and previous studies have shown that *Anopheles* mosquitoes avoid permanent habitats due to the presence, high abundance, and diversity of predators and competitor larvae thus suppressing the mosquito population densities [44]. Therefore, habitat productivity is also determined by the age of the habitat [45].

The low densities of *An. funestus* observed in the region [19] could be the reason behind the non-observance of *An. funestus* pupae during the study period. The high mortality rates of *An. arabiensis* immatures and *An. funestus* observed in the natural aquatic habitats is similar to previous findings in western Kenya [14], where larval survivorship was observed to be very low.

In summary, fish, dragonfly and water boatman were observed as efficient *Anopheles* larval predators. High larval mortality and low pupal productivity in larval habitats was also observed. A combination of efficient predation and high larval mortality will result in decreased adult malaria vector abundance and subsequently decreased malaria transmission in the area [46, 47]. The strength of this study was observed to be the comparison of the different predator types simultaneously and this provided a clear comparison of the larval predation rates amongst different classes of predators in a controlled environment. As a result, this study provided information on potential mosquito predators and their effectiveness. However, this study encountered a weakness of unavailability of species-specific predation rates within the predator classes as predation was only assessed at order or family level. This could result in considerable variation between species. There was also inability of reflect predation in natural conditions where mosquitoes may hide under aquatic vegetation or where the presence of other food sources for the predators may divert predators from mosquitoes. In addition, the biology and adaptations of certain predators, for example the odonates, which spend most of the time at the bottom while the *Anopheles* mosquitoes are found at the surface. This may affect their importance and capacity as predators.

In conclusion, the determination of the efficiency of various larval predators and habitat productivity will enhance the understanding of the mechanisms of mosquito larval density regulation and help with the correct identification of productive habitats and development of vector control methods that target productive habitats.

### Supplementary Information

**Additional file 1: Figure S1. **Correlation between larval densities (larvae per dip) and total number of predators for *Anopheles *larvae in (**A**) drainage ditches, (**B**) fishponds, (**C**) man-made ponds, and (**D**) rice paddies. **Figure S2. **Correlation between larval densities (larvae per dip) and total number of predators for *Culex *larvae in (**A**) drainage ditches, (**B**) fishponds, (**C**) man-made ponds, and (**D**) rice paddies. **Table S1.** Correlation between different predator types and larval densities/dip in the natural aquatic habitats.

## Data Availability

All data generated or analysed during this study are included in this published article [and its Additional file 1].

## References

[1] WHO (2020). The potential impact of health service disruptions on the burden of malaria: a modelling analysis for countries in sub-Saharan Africa.

[2] Maharaj R, Kissoon S, Lakan V, Kheswa N (2019). Rolling back malaria in Africa–challenges and opportunities to winning the elimination battle. S Afr Med J.

[3] Amimo F, Lambert B, Magit A, Sacarlal J, Hashizume M, Shibuya K (2020). *Plasmodium falciparum* resistance to sulfadoxine-pyrimethamine in Africa: a systematic analysis of national trends. BMJ Glob Health.

[4] Lai S, Sun J, Ruktanonchai NW, Zhou S, Yu J, Routledge I (2019). Changing epidemiology and challenges of malaria in China towards elimination. Malar J.

[5] Lindsay SW, Thomas MB, Kleinschmidt I (2021). Threats to the effectiveness of insecticide-treated bednets for malaria control: thinking beyond insecticide resistance. Lancet Glob Health.

[6] Zinszer K, Talisuna AO (2023). Fighting insecticide resistance in malaria control. Lancet Infect Dis.

[7] WHO (2018). First malaria vaccine in Africa: a potential new tool for child health and improved malaria control.

[8] Hogan AB, Winskill P, Ghani AC (2020). Estimated impact of RTS, S/AS01 malaria vaccine allocation strategies in sub-Saharan Africa: a modelling study. PLoS Med.

[9] Antonio-Nkondjio C, Sandjo NN, Awono-Ambene P, Wondji CS (2018). Implementing a larviciding efficacy or effectiveness control intervention against malaria vectors: key parameters for success. Parasit Vectors.

[10] Zhou G, Lo E, Githeko AK, Afrane YA, Yan G (2020). Long-lasting microbial larvicides for controlling insecticide resistant and outdoor transmitting vectors: a cost-effective supplement for malaria interventions. Infect Dis Poverty.

[11] Kahindi SC, Muriu S, Derua YA, Wang X, Zhou G, Lee MC (2018). Efficacy and persistence of long-lasting microbial larvicides against malaria vectors in western Kenya highlands. Parasit Vectors.

[12] Orondo PW, Wang X, Lee MC, Nyanjom SG, Atieli H, Ondeto BM (2023). Habitat diversity, stability, and productivity of malaria vectors in irrigated and nonirrigated ecosystems in Western Kenya. J Med Entomol.

[13] Kweka EJ, Zhou G, Gilbreath TM, Afrane Y, Nyindo M, Githeko AK (2011). Predation efficiency of *Anopheles gambiae* larvae by aquatic predators in western Kenya highlands. Parasit Vectors.

[14] Munga S, Minakawa N, Zhou G, Githeko AK, Yan G (2007). Survivorship of immature stages of *Anopheles gambiae* s.l. (Diptera: Culicidae) in natural habitats in western Kenya highlands. J Med Entomol.

[15] Dambach P (2020). The use of aquatic predators for larval control of mosquito disease vectors: opportunities and limitations. Biol Control.

[16] Carlson J, Keating J, Mbogo CM, Kahindi S, Beier JC (2004). Ecological limitations on aquatic mosquito predator colonization in the urban environment. J Vector Ecol.

[17] Lyimo EO, Takken W, Koella JC (1992). Effect of rearing temperature and larval density on larval survival, age at pupation and adult size of *Anopheles gambiae*. Entomol Exp Applicata.

[18] Makone SM, Basweti EA, Bunyatta DK (2021). Effects of irrigation systems on farming practices: evidence from oluch-kimira scheme, Homa Bay County Kenya. Asian J Adv Res Rep.

[19] Ondeto BM, Wang X, Atieli H, Orondo PW, Ochwedo KO, Omondi CJ (2022). Malaria vector bionomics and transmission in irrigated and non-irrigated sites in western Kenya. Parasitol Res.

[20] Omondi CJ, Otambo WO, Odongo D, Ochwedo KO, Otieno A, Onyango SA (2022). Asymptomatic and submicroscopic *Plasmodium* infections in an area before and during integrated vector control in Homa Bay, western Kenya. Malar J.

[21] Allo NM, Mekhlif AF (2019). Role of the predator *Anisops*
*sardea* (Hemiptera: Notonectidae) in control mosquito *Culex*
*pipiens* molestus (Diptera: Culicidae) population. Int J Mosq Res.

[22] Reisen WK, Siddiqui TF (1979). Horizontal and vertical estimates of immature survivorship for *Culex*
*tritaeniorhynchus* (Diptera: Culicidae) in Pakistan. J Med Entomol.

[23] Edillo FE, Touré YT, Lanzaro GC, Dolo G, Taylor CE (2004). Survivorship and distribution of immature *Anopheles*
*gambiae* s.l. (Diptera: Culicidae) in Banambani village Mali. J Med Entomol.

[24] Okogun GR (2005). Life-table analysis of *Anopheles* malaria vectors: generational mortality as tool in mosquito vector abundance and control studies. J Vector Borne Dis.

[25] Shaalan EA, Canyon DV (2009). Aquatic insect predators and mosquito control. Trop Biomed.

[26] Ranasinghe HA, Amarasinghe LD (2020). Naturally occurring microbiota associated with mosquito breeding habitats and their effects on mosquito larvae. Biomed Res Int.

[27] Quiroz-Martínez H, Rodríguez-Castro A (2007). Aquatic insects as predators of mosquito larvae. J Am Mosq Control Assoc.

[28] Acquah-Lamptey D, Brandl R (2018). Effect of a dragonfly (*Bradinopyga*
*strachani* Kirby, 1900) on the density of mosquito larvae in a field experiment using mesocosms. Web Ecol.

[29] Sunahara T, Ishizaka K, Mogi M (2002). Habitat size: a factor determining the opportunity for encounters between mosquito larvae and aquatic predators. J Vector Ecol.

[30] Minakawa N, Sonye G, Mogi M, Yan G (2004). Habitat characteristics of *Anopheles gambiae* s.s. larvae in a Kenyan highland. Med Vet Entomol.

[31] Kiflawi M, Blaustein L, Mangel M (2003). Oviposition habitat selection by the mosquito *Culiseta*
*longiareolata* in response to risk of predation and conspecific larval density. Ecol Entomol.

[32] Blaustein L, Kiflawi M, Eitam A, Mangel M, Cohen JE (2004). Oviposition habitat selection in response to risk of predation in temporary pools: mode of detection and consistency across experimental venue. Oecologia.

[33] Sumba LA, Guda TO, Deng AL, Hassanali A, Beier JC, Knols BG (2004). Mediation of oviposition site selection in the African malaria mosquito *Anopheles gambiae* (Diptera: Culicidae) by semiochemicals of microbial origin. Int J Trop Insect Sci.

[34] Shaukat MA, Ali S, Saddiq B, Hassan MW, Ahmad A, Kamran M (2019). Effective mechanisms to control mosquito borne diseases: a review. Am J Clin Neurol Neurosurg.

[35] Scott MA, Murdoch WW (1983). Selective predation by the backswimmer, *Notonecta*1. Limnol Oceanogr.

[36] Notestine MK (1971). Population densities of known invertebrate predators of mosquito larvae in Utah marshlands. Mosq News.

[37] Eba K, Duchateau L, Olkeba BK, Boets P, Bedada D, Goethals PL (2021). Bio-control of *Anopheles* mosquito larvae using invertebrate predators to support human health programs in Ethiopia. Int J Environ Res Public Health.

[38] Buxton M, Cuthbert RN, Dalu T, Nyamukondiwa C, Wasserman RJ (2020). Complementary impacts of heterospecific predators facilitate improved biological control of mosquito larvae. Biol Control.

[39] Mohammed SH, Eltaly RI, Salem HH (2022). Toxicological and biochemical studies for chlorpyrifos insecticide on some mosquito larvae and their associated predators. Egyptian J Basic Appl Sci.

[40] Dida GO, Gelder FB, Anyona DN, Abuom PO, Onyuka JO, Matano AS (2015). Presence and distribution of mosquito larvae predators and factors influencing their abundance along the Mara River Kenya and Tanzania. Springerplus.

[41] Munga S, Minakawa N, Zhou G, Barrack OO, Githeko AK, Yan G (2014). Effects of larval competitors and predators on oviposition site selection of *Anopheles gambiae* sensu stricto. J Med Entomol.

[42] Louca V, Lucas MC, Green C, Majambere S, Fillinger U, Lindsay SW (2014). Role of fish as predators of mosquito larvae on the floodplain of the Gambia River. J Med Entomol.

[43] Zuharah WF, Lester PJ (2010). The influence of aquatic predators on mosquito abundance in animal drinking troughs in New Zealand. J Vector Ecol.

[44] Mereta ST, Yewhalaw D, Boets P, Ahmed A, Duchateau L, Speybroeck N (2013). Physico-chemical and biological characterization of anopheline mosquito larval habitats (Diptera: Culicidae): implications for malaria control. Parasit Vectors.

[45] Munga S, Vulule J, Kweka EJ (2013). Response of *Anopheles gambiae* s.l. (Diptera: Culicidae) to larval habitat age in western Kenya highlands. Parasit Vectors.

[46] Otambo WO, Onyango PO, Wang C, Olumeh J, Ondeto BM, Lee MC (2022). Influence of landscape heterogeneity on entomological and parasitological indices of malaria in Kisumu Western Kenya. Parasit Vectors.

[47] Githeko AK, Ayisi JM, Odada PK, Atieli FK, Ndenga BA, Githure JI (2006). Topography and malaria transmission heterogeneity in western Kenya highlands: prospects for focal vector control. Malar J.

