# Supplementary material for: Effect of predators on Anopheles arabiensis and Anopheles funestus larval survivorship in Homa Bay County Western Kenya
Source: Malar J. 2023 Oct 5;22:298. doi: 10.1186/s12936-023-04741-w (PMC10557226; doi:10.1186/s12936-023-04741-w)

**SUPPLEMENTARY FILE**

**Table S1:** Correlation between different predator types and larval densities/ dip in the natural aquatic habitats.

| **Predator Type** | ***Anopheles* larval densities/ dip** | ***Culex* larval densities/ dip** |
| --- | --- | --- |
| Fish | -0.19645 | 0.000734 |
| Amphibians | 0.166921 | -0.06615 |
| Annelids | -0.06884 | -0.24966 |
| Arachnids | -0.15556 | -0.08607 |
| Coleoptera | -0.01431 | -0.18576 |
| Diptera | -0.02784 | -0.03196 |
| Ephmeroptera | -0.13557 | -0.02479 |
| Molluscs | -0.16688 | -0.00397 |
| Odonata | -0.20083 | -0.27104 |
| Hemiptera | 0.023883 | -0.13926 |
| Other Predators | -0.00865 | 0.087648 |


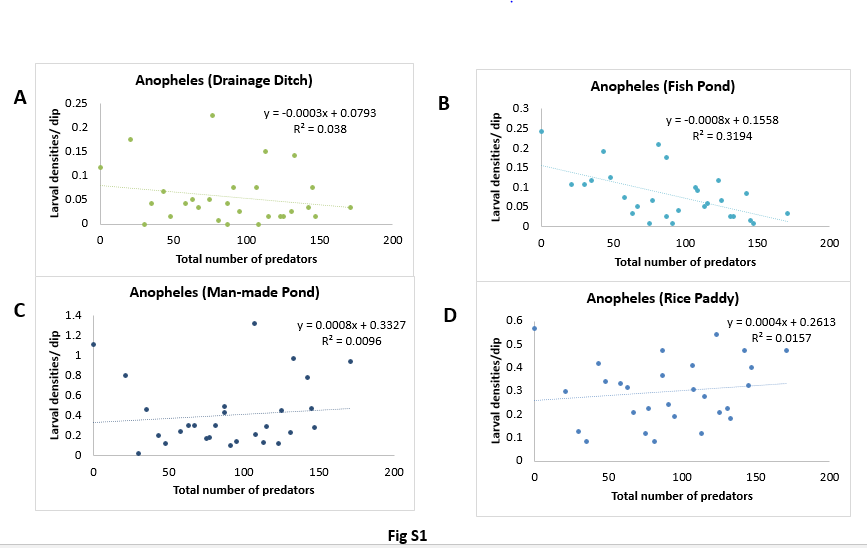


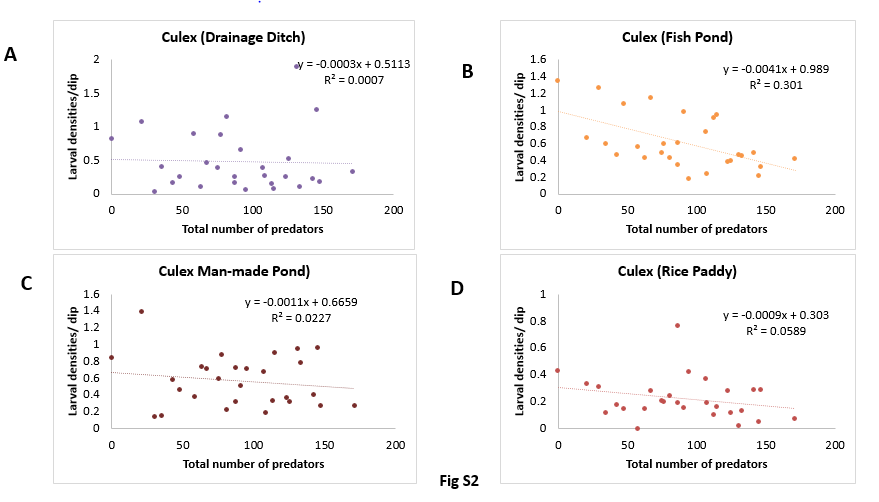

Supplement: Supplementary file 1 — Additional file 1: Figure S1. Correlation between larval densities (larvae per dip) and total number of predators for Anopheles larvae in (A) drainage ditches, (B) fishponds, (C) man-made ponds, and (D) rice paddies. Figure S2. Correlation between larval densities (larvae per dip) and total number of predators for Culex larvae in (A) drainage ditches, (B) fishponds, (C) man-made ponds, and (D) rice paddies. Table S1. Correlation between different predator types and larval densities/dip in the natural aquatic habitats. [file 12936_2023_4741_MOESM1_ESM.docx]
